# Longitudinal variability of lipoprotein(a) in youth-onset type 1 diabetes: implications for cardiovascular risk stratification

**DOI:** 10.1186/s12933-026-03252-7

**Published:** 2026-06-19

**Authors:** Fanny Iafrate-Luterbacher, Cecilia Jimenez-Sanchez, Maria Loukia Anastasiadou, Julien Prados, Frida Renström, Michael Brändle, Stefan Bilz, Valerie M. Schwitzgebel

**Affiliations:** 1 Division of General Paediatrics, Department of Paediatrics, Gynaecology and Obstetrics, University Hospitals of Geneva, Geneva, Switzerland; 2 Pediatric Endocrine and Diabetes Unit, Division of Development and Growth, Department of Paediatrics, Gynaecology and Obstetrics, University Hospitals of Geneva, Rue Willy-Donzé 6, 1205 Geneve, Switzerland; 3 Bioinformatics Support Platform, University of Geneva, Geneva, Switzerland; 4 Division of Endocrinology and Diabetes, Department of Internal Medicine, Cantonal Hospital St. Gallen, HOCH Health Ostschweiz, St. Gallen, Switzerland; 5 Diabetes Centre of the Faculty of Medicine, University of Geneva, Geneva, Switzerland; 6 Institute of Genetics and Genomics (iGE3) in Geneva, University of Geneva, 1211 Geneva, Switzerland

**Keywords:** Cardiovascular risk, Adolescence, Dyslipidaemia, Biomarker variability, Coronary artery disease, Cardiovascular disease, Diabetes complications, Hypercholesterolaemia, Macrovascular complications, Paediatric diabetes

## Abstract

**Background:**

Lipoprotein(a) [Lp(a)] is a genetically determined and independent cardiovascular risk factor, traditionally considered stable across the lifespan, supporting a single lifetime measurement strategy. However, its longitudinal behaviour during childhood and adolescence remains poorly characterised, particularly in individuals with type 1 diabetes who face a markedly increased lifetime risk of coronary artery disease. We therefore aimed to characterise intra- and inter-individual trajectories of Lp(a) in a paediatric type 1 diabetes cohort and to assess the implications of Lp(a) variability for cardiovascular risk classification.

**Methods:**

We conducted a retrospective single-centre cohort study of children and adolescents with type 1 diabetes attending Geneva University Hospitals between 2012 and 2023. Annual fasting Lp(a) concentrations were analysed longitudinally. Variability was assessed in participants with ≥ 2 measurements. Clinically relevant thresholds were used to evaluate cardiovascular risk reclassification. Paired Wilcoxon tests, Pearson and Kendall correlations, and Holm-adjusted *p*-values (*P* < 0.05) were applied. Analyses were conducted in R.

**Results:**

A total of 286 participants contributed 1403 Lp(a) measurements, with observation periods varying across individuals (median 6.2 years, IQR 2.9–9.6) and between 1 and 13 measurements per participant. At baseline, 26% had elevated Lp(a) (≥ 300 mg/l). Among participants with serial measurements, 32% showed intraindividual fluctuations exceeding 50% of their individual maximum value. Reclassification across the 300 mg/l cardiovascular risk threshold occurred in 11.9% of participants. Lp(a) concentrations peaked between ages 10 and 13 years and declined thereafter. Modest seasonal variation was observed, with higher concentrations in autumn and winter (*P* < 0.05).

**Conclusions:**

In youth with type 1 diabetes, Lp(a) is not as stable as previously assumed, exhibiting clinically relevant variability over time. These findings challenge the current paradigm of a single lifetime Lp(a) measurement and suggest that repeated assessment, particularly during adolescence, may improve early cardiovascular risk stratification.

**Graphical abstract:**

**Figure:**
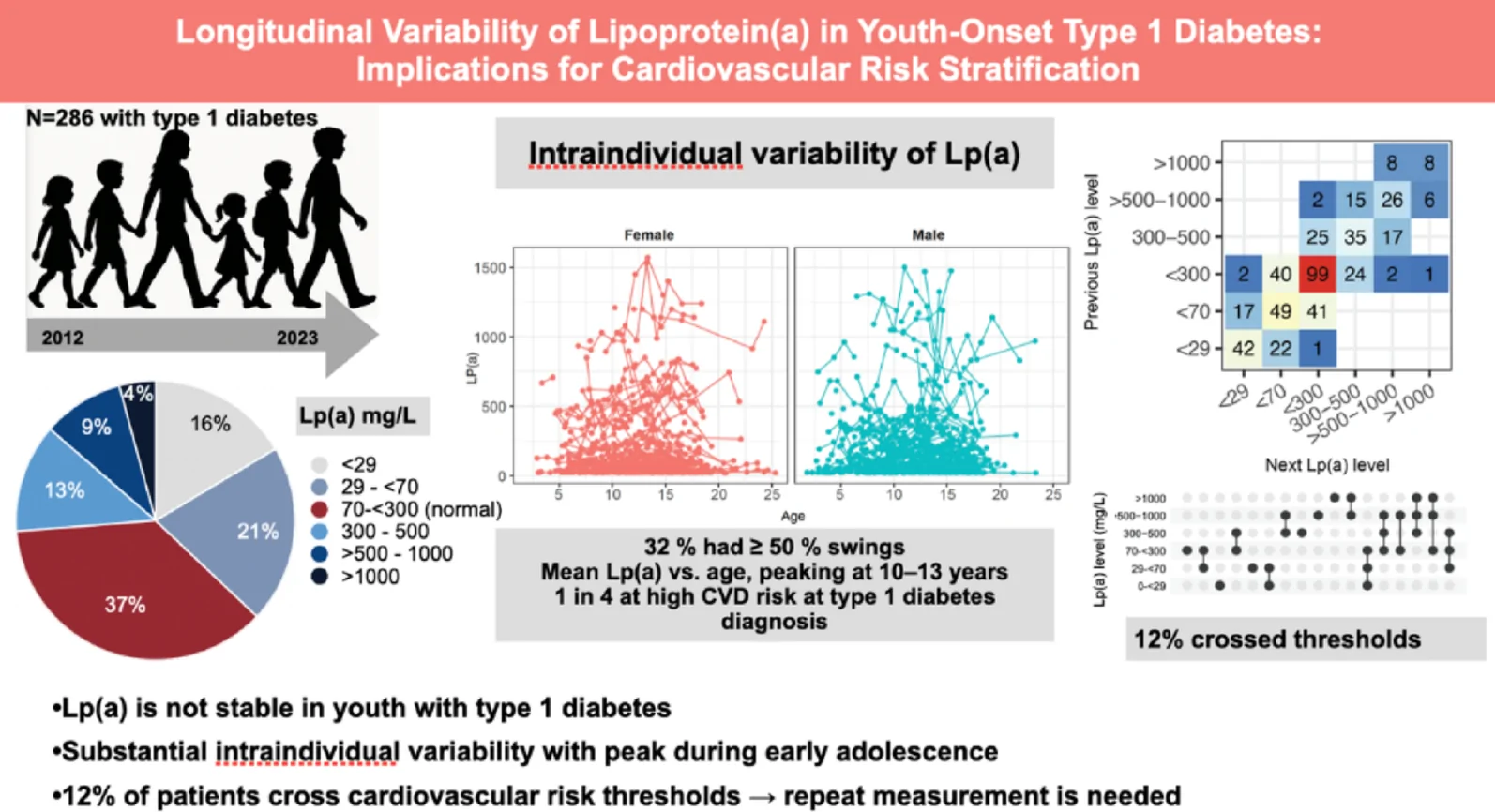

**Supplementary Information:**

The online version contains supplementary material available at https://doi.org/10.1186/s12933-026-03252-7.

## Research insight

**What is currently known about this topic?**

Children with type 1 diabetes (T1D) have a markedly increased lifetime risk of cardiovascular disease (CVD). Elevated lipoprotein(a) [Lp(a)] is an established predictor of atherosclerotic outcomes, but its longitudinal behaviour in paediatric T1D is poorly understood.

**What is the key research question?**

Does Lp(a) vary within individuals during childhood in T1D, and does this variability affect CVD risk classification?

**What is new?**

Lp(a) levels in youth with T1D exhibit substantial intraindividual variability, with a peak in early adolescence and modest seasonal fluctuations. This variability results in clinically meaningful reclassification of CVD risk in a significant proportion of patients.

**How might this study influence clinical practice?**

These findings challenge the current paradigm of a single lifetime Lp(a) measurement and support repeated assessment to improve CVD risk stratification and guide earlier preventive interventions.

## Introduction

Lipoprotein(a) [Lp(a)] has attracted growing interest as a genetically determined independent risk factor for cardiovascular disease (CVD) [1, 2]. Structurally analogous to low-density lipoprotein (LDL), Lp(a) consists of an apolipoprotein B-100 particle covalently linked to apolipoprotein(a), which carries proatherogenic oxidized phospholipids [3]. Elevated Lp(a) promotes atherogenesis through pro-inflammatory, pro-thrombotic, and pro-oxidative mechanisms, impairing fibrinolysis and contributing to plaque instability [4]. Genome-wide association studies consistently identify *LPA* variants as the strongest genetic determinants of CAD, with each genetically mediated one-standard-deviation reduction in Lp(a) corresponding to a 29% lower coronary heart disease risk [5]. Clinically, Lp(a) levels above 300 mg/L confer increased CVD risk [6].

Children with early-onset type 1 diabetes face a markedly increased lifetime risk of CVD, with coronary artery disease (CAD) emerging much earlier than in the general population [7]. Of clinical concern, subclinical cardiovascular abnormalities, including dysregulated lipoprotein profiles, elevated inflammatory cytokines, and endothelial dysfunction, can already be detected during childhood and adolescence, establishing a foundation for heightened lifetime risk [7].

Given its strong genetic basis, Lp(a) is traditionally considered stable across the lifespan, leading to the recommendation of a single lifetime measurement [6, 8]. However, this assumption is largely based on adult data, and evidence in paediatric populations, particularly in high-risk groups such as individuals with type 1 diabetes, remains scarce.

Importantly, emerging Lp(a)-lowering therapies, including antisense oligonucleotides and small interfering RNAs directed against apolipoprotein(a), have demonstrated substantial and sustained reductions in circulating Lp(a) levels in adult populations [9]. These advances highlight the growing clinical relevance of accurately identifying individuals at risk early in life.

In this context, characterizing the longitudinal behaviour of Lp(a) and quantifying the extent of its intraindividual variability during childhood and adolescence is critical. We therefore aimed to describe intra- and inter-individual trajectories of Lp(a) in a longitudinal cohort of youth with type 1 diabetes and to evaluate the implications of this variability for cardiovascular risk stratification.

## Methods

We conducted a retrospective, single-centre study at Geneva University Hospitals analysing fasting Lp(a) annually from 2012 to 2023 (*n* = 1403 measurements). To be eligible, patients had to meet the following criteria: diagnosis of type 1 diabetes, at least one Lp(a) measurement, a minimum of one year of follow-up by the Geneva paediatric diabetes unit, and age under 25 years. In total, 286 patients met these criteria and comprised the full study cohort. A subgroup of 235 patients who had at least two Lp(a) measurements was used for the analysis of intraindividual variability.

Yearly blood analyses were performed in the morning after an overnight fast. Annual blood draws were performed only when participants were clinically well and postponed in case of acute illness (e.g., fever or viral infection). Data on demographic characteristics (age, sex, BMI), insulin dose, and laboratory measurements including Lp(a), haemoglobin A1c, triglycerides, total cholesterol, HDL cholesterol, and LDL cholesterol, thyroid function tests as well as anti-thyroperoxidase, anti-thyroglobulin and anti-transglutaminase IgA antibodies with total IgA were collected at diagnosis and after each annual clinical assessment. The samples were analysed in real time. The REDCap platform was used for data collection [10]. All Lp(a) measurements over 2012–2023 were performed using an immunoturbidimetric method with a coefficient of variation below 8.3% (Roche Diagnostics SA No art. 05852625 190, instrument: Roche Cobas 8000), which is isoform insensitive. Internal quality control procedures were performed regularly.

### Statistical methods

We performed descriptive analyses for all study variables. For continuous variables, means, SDs, and medians were calculated; frequency counts and percentages were calculated for categorical variables. Lp(a) values were log-transformed to reduce right skew in the distribution. To assess the association between Lp(a) levels and predefined factors (BMI, ethnicity, glycated haemoglobin, daily insulin dose, sex, age), we used two-sample Wilcoxon tests for categorical variables and Kendall or Pearson correlation tests for continuous variables, depending on data distribution. When appropriate, p-values were adjusted for multiple testing using Holm’s method. Statistical significance was set at *P* < 0.05 for all analyses. To analyse intraindividual variability in Lp(a) concentrations over time, we restricted the analysis to 235 patients with at least two annual assessments. Changes in Lp(a) levels between consecutive visits were quantified. For the seasonality analysis, changes in Lp(a) were calculated relative to each patient’s median Lp(a) level across visits. All preprocessing, analysis, and visualisation were performed using the R programming language [R: A Language and Environment for Statistical Computing; R Core Team; https://www.R-project.org/, 2025].

### Risk curves

To estimate the risk for a patient to reach a pathological Lp(a) levels of 300 mg/L (resp. 500 mg/L and 1000 mg/L) given an observed Lp(a) level x, we computed from our data the ratio M(x)/N(x), where N(x) is the number of patient whose range of variation of Lp(a) include the value x, while M(x) is the number of patient whose range of variation of Lp(a) include both x and 300 (resp. 500 mg/l and 1000 mg/L).

## Results

**Fig. 1:**
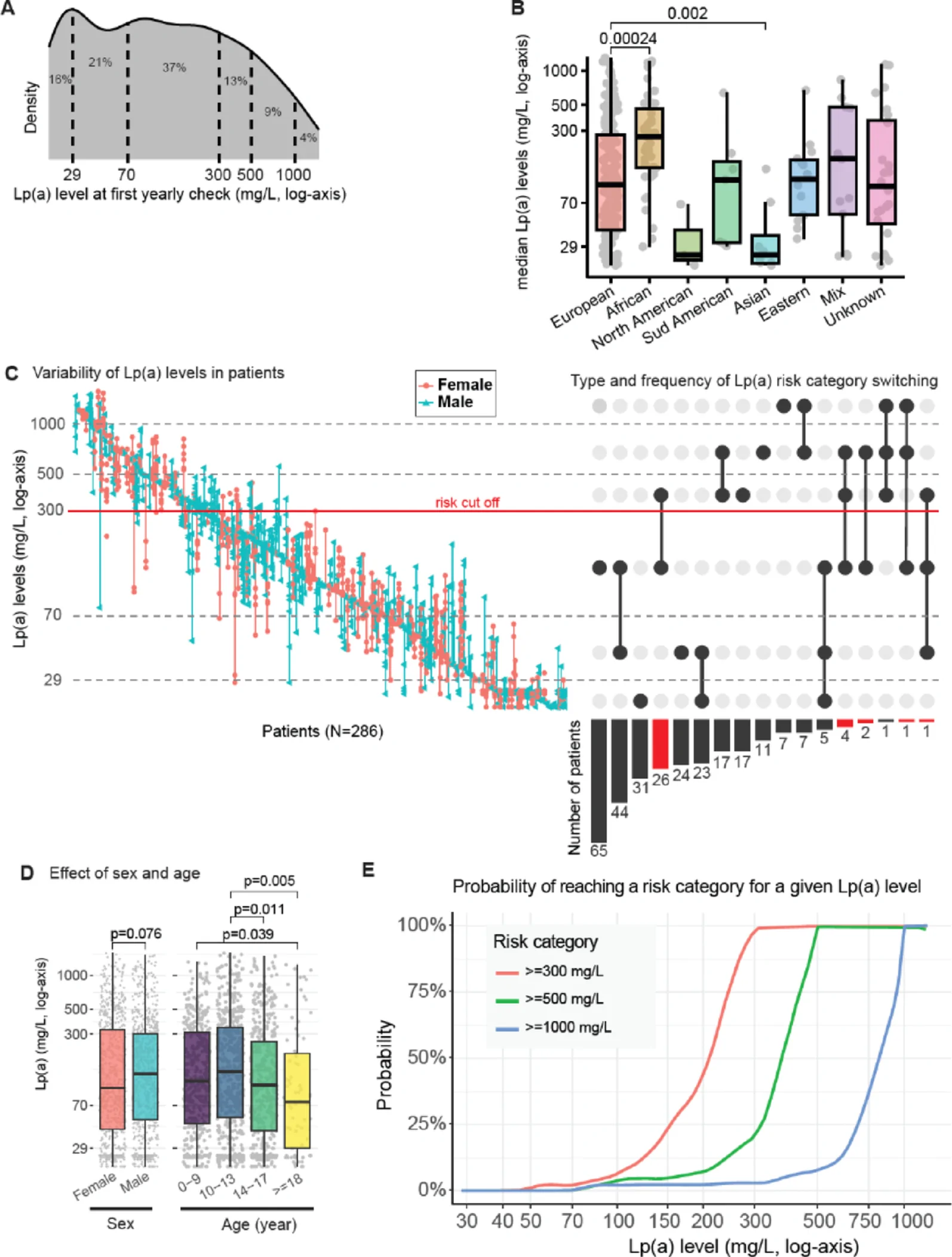
Longitudinal variability and cardiovascular risk stratification of Lp(a) in youth with type 1 diabetes. *Does Lp(a) vary clinically meaningfully in this population, and does this variability affect cardiovascular risk classification?* **A** Baseline Lp(a) distribution. Density distribution of Lp(a) at first measurement (log-scale), with dashed lines indicating established cardiovascular risk thresholds. More than a quarter of participants (26%) already exceeded the 300 mg/L risk threshold at baseline, and 4% exceeded 1,000 mg/L. **B** Ancestry-related variation. Median Lp(a) levels by patient ancestry. Children of African descent had significantly higher Lp(a), and East Asian children significantly lower Lp(a), compared with European peers, consistent with known genetic determinants. Pairwise comparisons used Wilcoxon tests with Holm adjustment; only *P* ≤ 0.05 are shown. **C** Intra-individual variability and risk reclassification. Left: individual Lp(a) trajectories across all visits, sorted by each patient's median value; females in red points, males as teal triangles. Both upward and downward transitions are visible, with no dominant linear trend. Right: UpSet plot quantifying risk category transitions over time. Clinically relevant reclassification across the 300 mg/L threshold occurred in 11.9% of participants (shown in red), demonstrating that a single measurement can misclassify a significant proportion of patients. **D** Age- and sex-related trajectory. Boxplots of all 1,403 Lp(a) measurements stratified by age group and sex. Lp(a) peaked between ages 10 and 13 years and declined in late adolescence, with no significant sex difference (*P* = 0.076). This pattern suggests a developmental influence on Lp(a) during puberty. Wilcoxon tests with Holm adjustment; only *P* ≤ 0.05 are shown. **E** Probability of future threshold crossing. Risk curves estimating the probability that a future Lp(a) measurement will exceed the 300, 500, or 1,000 mg/L thresholds, given a known baseline value. A patient with a low baseline Lp(a) is unlikely, but not certain, to remain below risk thresholds, providing a practical tool for individualised longitudinal risk assessment

Of 435 eligible patients invited, 286 children and adolescents with type 1 diabetes consented to participate, contributing between 1 and 13 Lp(a) measurements per participant (median 5) over a median follow-up of 6.2 years (IQR 2.9–9.6). Baseline characteristics are summarized in the supplementary Table 1. Figure 1 provides a comprehensive overview of Lp(a) distribution. Lp(a) concentrations were stratified into six risk categories based on established CAD risk thresholds (Fig. 1A), with 300 mg/L denoting elevated cardiovascular risk [8]. We show that more than a quarter of participants already exceeded the 300 mg/L cardiovascular risk threshold at baseline. At the first measurement, 26% of participants exhibited elevated Lp(a) (≥ 300 mg/L), with 4% exceeding 1,000 mg/L. Thirty-seven percent displayed low (< 70 mg/L) or undetectable (< 29 mg/L) values, and 37% fell within the reference range (70–< 300 mg/L) (Fig. 1A). Lp(a) levels showed a mild correlation with HbA1c (Fig. S1), but not with sex, age at diabetes onset, BMI, or insulin dose (Additional file 1). We further analysed ancestry-related variation. Lp(a) levels varied significantly by ancestry: children of African descent exhibited higher values, whereas East Asian children had significantly lower Lp(a) compared with their European peers (Fig. 1B). We further characterised intraindividual Lp(a) variability over time (Fig. 1C).

Among 235 patients with at least two measurements, 32% demonstrated fluctuations exceeding 50% of their personal maximum Lp(a) value (Fig. 1C and Fig. S2 with table). This proportion remained stable when the analysis was restricted to patients with a personal maximum Lp(a) ≥ 300 mg/L (35%), confirming that clinically meaningful variability is not driven by analytical noise at low absolute values. Variability patterns were both uni- and bidirectional across consecutive visits, with no simple linear trend over time. The extent of risk category reclassification is illustrated in the accompanying UpSet plot (Fig. 1C). Clinically meaningful reclassification across the 300 mg/L threshold occurred in 11.9% of patients (shown in red): 9% transitioned between normal and high-risk categories, while 2.4% shifted between no risk and very high risk (> 1,000 mg/L). Variability was present in both sexes. To assess whether the presence of additional autoimmune conditions influenced intraindividual Lp(a) variability, we stratified the variability subgroup by the presence of Hashimoto thyroiditis or coeliac disease. The proportion of patients with a ≥ twofold intraindividual Lp(a) range was comparable between those without (32%, 69/214) and those with (28%, 6/21) a co-occurring autoimmune condition (Fig. S2), indicating that autoimmune comorbidities do not substantially contribute to the variability observed in this cohort.

In panel D we illustrate the age- and sex-stratified distribution of all 1,403 measurements, revealing a clear peak in Lp(a) concentrations between 10 and 13 years of age followed by a decline in late adolescence, with no significant difference between sexes (Fig. 1D). We modelled risk curves, showing how baseline Lp(a) values can be used to estimate the probability of a future measurement exceeding the 300, 500, or 1,000 mg/L thresholds, offering a practical tool for individualised longitudinal risk assessment (Fig. 1E).

**Fig. 2:**
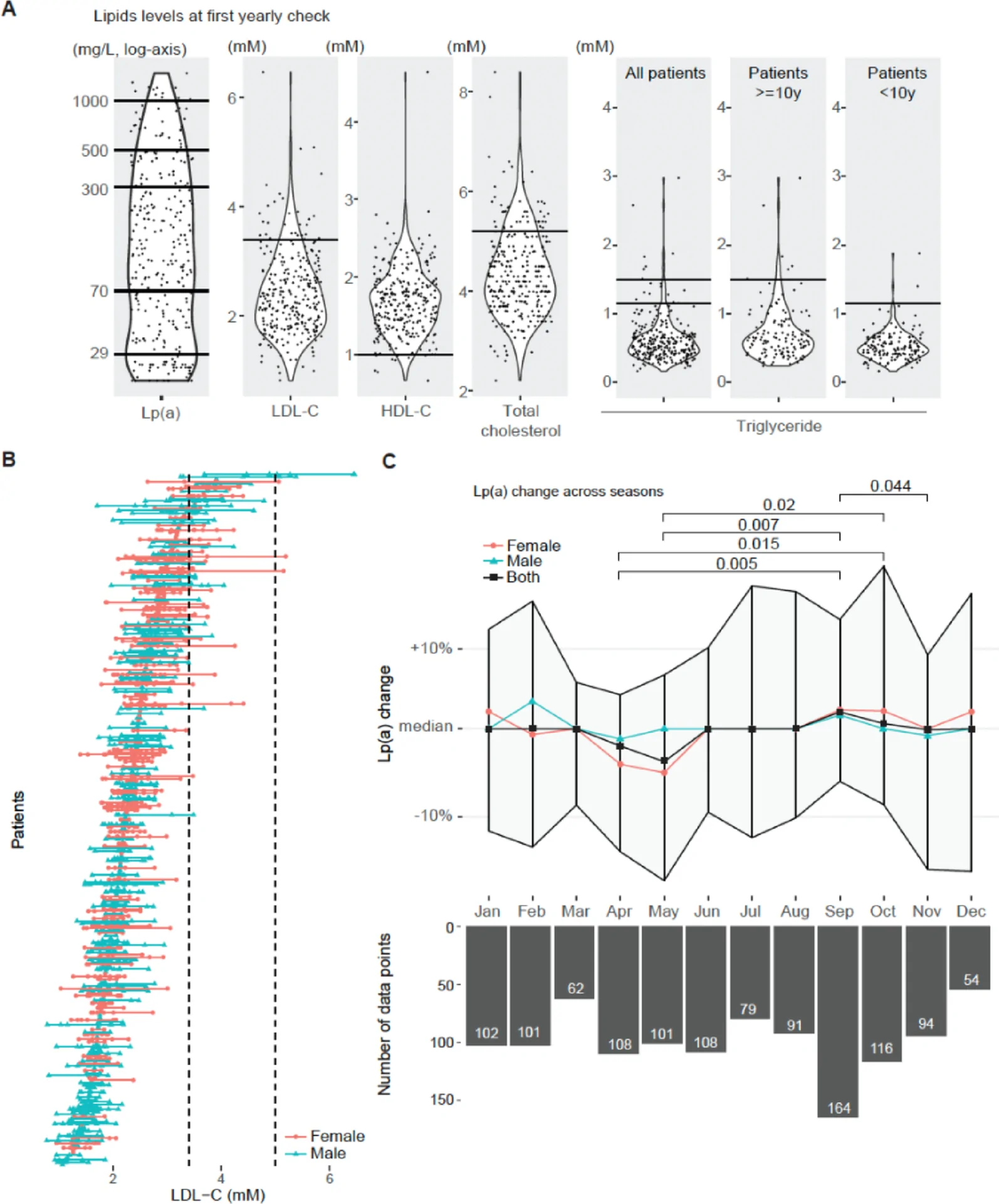
Lipid profiles and seasonal variation of Lp(a) in youth with type 1 diabetes. *Are Lp(a) elevations accompanied by other lipid abnormalities, and is there evidence of environmental modulation of Lp(a) over time?* **A** Lipid profile at first annual assessment. Violin plots of Lp(a) (log-scale) and standard lipid parameters. Clinically relevant thresholds are indicated by horizontal lines. At the first annual assessment, elevated LDL-C (> 3.4 mM) was present in 10% of participants. Of these, 5 participants (1.7%) had LDL-C > 5 mM and 14 (4.9%) had levels between 4 and 5 mM at any point during follow-up, prompting further clinical evaluation for familial hypercholesterolaemia. **B** Inter-individual LDL-C variability. LDL-C levels for all participants, ordered by each patient's median LDL-C over time, illustrating the wide range of lipid profiles in this cohort. **C** Seasonal variation in Lp(a). Lp(a) was modestly but significantly higher during autumn and winter relative to spring and summer (*n* = 1,180 measurements from 167 patients with ≥ 4 assessments). Values represent percent change relative to each patient's own median. The black line shows the median trend; red and blue lines show sex-specific trends; shaded areas represent IQRs

At the first annual review, 78% of participants displayed no fasting dyslipidaemia. Elevated LDL-C (> 3.4 mM) was observed in 10%, with 1.7% above 5 mM (Fig. 2A). LDL-C variability occurred in both sexes (Fig. 2B). Notably, 9% of the cohort had concomitantly elevated Lp(a) and LDL-C, a combination known to significantly amplify CAD risk [11]. This subgroup may represent individuals at particularly high cardiovascular risk, potentially including cases of undiagnosed familial hypercholesterolaemia.

In 167 individuals with four or more Lp(a) measurements (*n* = 1,180), seasonal variation was detected. Lp(a) were modestly but significantly higher during autumn and winter relative to spring and summer, with the largest monthly differences observed between April and September (Fig. 2C). Changes were calculated relative to each patient’s median Lp(a) level across visits.

## Discussion

In this longitudinal paediatric type 1 diabetes cohort, we show that Lp(a), traditionally considered a genetically determined and stable biomarker, exhibits substantial intraindividual variability across childhood and adolescence. More than 11% of participants crossed clinically relevant cardiovascular risk thresholds over time, highlighting the potential for misclassification when relying on a single measurement.

Beyond its descriptive relevance, this variability may also have biological and clinical implications. Analogous to glycaemic or lipid variability, fluctuations in Lp(a) could contribute to vascular damage through intermittent exposure to pro-atherogenic lipoprotein profiles, promoting oxidative stress, endothelial dysfunction, and low-grade inflammation, key processes in early atherogenesis [12, 13].

We observed a distinct age-related trajectory, with Lp(a) levels peaking between 10 and 13 years and declining thereafter. This pattern suggests a potential influence of pubertal hormonal changes, including sex steroids and growth factor signalling, on hepatic Lp(a) production [14]. Adolescence may therefore represent a critical window during which Lp(a)-based cardiovascular risk assessment is particularly informative.

The age-related trajectory we describe is broadly consistent with observations from general paediatric cohorts. Raitakari and colleagues, in a large longitudinal study of youth without diabetes, demonstrated that Lp(a) concentrations measured in childhood and adolescence track into adulthood and independently predict major cardiovascular outcomes, with levels showing a degree of developmental variation across age strata [2]. Our finding of a peak in early adolescence followed by a gradual decline aligns with the hypothesis that pubertal hormonal signalling modulates hepatic apolipoprotein(a) production transiently, a pattern that does not appear to be unique to metabolically high-risk populations [15]. Absolute Lp(a) concentrations in our cohort were broadly comparable to those reported in non-diabetic paediatric populations, including NHANES-III (median 70–120 mg/L in non-Hispanic White children aged 4–19 years [16]) and the Bogalusa Heart Study [15], suggesting that type 1 diabetes does not substantially elevate baseline Lp(a), though a modest glycaemic influence cannot be excluded given the mild HbA1c correlation observed. However, because our cohort comprises exclusively children and adolescents with type 1 diabetes and lacks a contemporaneously measured non-diabetic comparator group, we cannot formally determine whether diabetes-related metabolic perturbations, including chronic subclinical inflammation, insulin-mediated hepatic signalling, or glycaemic variability amplify or prolong this developmental peak beyond what would be expected in the general paediatric population. The possibility of an incremental type 1 diabetes-specific effect on Lp(a) trajectories therefore remains plausible but unresolved by the present data and warrants direct investigation in future studies with paired diabetic and non-diabetic control cohorts. Taken together, these observations suggest that Lp(a) concentrations during childhood and adolescence may be more responsive to developmental and metabolic influences than the traditional view of Lp(a) as a largely genetically fixed trait would imply, without precluding the possibility that much of the within-individual variation we observed reflects the normal biology of Lp(a) across growth and puberty.

Seasonal variation further supports the notion that Lp(a) is not entirely biologically inert. Higher levels during autumn and winter may reflect inflammatory modulation, potentially mediated by cytokines such as interleukin-6, as well as environmental influences including vitamin D status and dietary patterns [14, 17]. Although the magnitude of these differences was modest, they reinforce the concept that both developmental and environmental factors can influence Lp(a) concentrations.

Importantly, a subset of patients exhibited concomitant elevations in Lp(a) and LDL-C, a lipid profile associated with markedly increased cardiovascular risk [11]. Since Lp(a)-associated cholesterol contributes to measured LDL-C levels, this overlap may complicate risk stratification and potentially mask conditions such as familial hypercholesterolaemia [18, 19]. These findings support the integration of Lp(a) into routine lipid assessment in paediatric diabetes populations.

Ethnic differences observed in our cohort are consistent with previous reports, underscoring the need for ancestry-specific reference ranges and a more personalised approach to cardiovascular risk stratification [20, 21].

Taken together, our findings suggest that Lp(a) concentrations in young patients with type 1 diabetes exhibit clinically relevant temporal intraindividual variability. These observations support the potential value of longitudinal assessment, particularly during key developmental stages.

### Strengths and limitations

The main strength of this study lies in its longitudinal design, with repeated annual measurements of Lp(a) over a median follow-up exceeding six years, enabling robust assessment of intraindividual variability across key developmental stages. The use of an isoform-insensitive immunoturbidimetric assay further strengthens the reliability of Lp(a) quantification across a wide range of concentrations. In addition, the comprehensive clinical characterization of the cohort allowed exploration of associations with metabolic and demographic variables. However, several limitations should be acknowledged. This was a single-centre, retrospective study, which may limit generalisability to other populations with different genetic and environmental backgrounds. The absence of genetic data on *LPA* variants (kringle-IV) and of inflammatory biomarkers precludes mechanistic insights into the drivers of Lp(a) variability.

The study also lacks a contemporaneous non-diabetic paediatric control group. Although the age-related Lp(a) trajectory we describe with a peak in early adolescence followed by a decline is broadly consistent with data from general paediatric cohorts, the absence of a concurrently measured comparator group prevents us from formally determining whether the magnitude of variability or the timing of this peak reflects a pattern intrinsic to normal childhood development or an incremental effect of type 1 diabetes and its metabolic milieu. Although analytical variability cannot be excluded as a contributor to observed fluctuations, the magnitude of intraindividual variation substantially exceeds the assay coefficient of variation (< 8.3%), suggesting that true biological variability is the predominant driver. Finally, although we observed clinically relevant reclassification across risk thresholds, the study was not designed to capture clinical cardiovascular endpoints. One participant had overt hypothyroidism due to treatment non-compliance; although the stratified variability analysis showed comparable results between patients with and without autoimmune comorbidities, residual confounding from uncontrolled thyroid dysfunction in this individual cannot be entirely excluded.

## Conclusions

Lp(a) is not as stable as previously assumed in children and adolescents with type 1 diabetes, exhibiting substantial intraindividual variability and clinically relevant shifts in cardiovascular risk classification over time. These findings challenge the current paradigm of a single lifetime Lp(a) measurement and support the need for repeated assessment, particularly during adolescence. A longitudinal approach to Lp(a) monitoring may improve early cardiovascular risk stratification and inform personalised preventive strategies in this high-risk population.

## Supplementary Information


Supplementary Material 1.


## Data Availability

Deidentified data are available from the corresponding author upon reasonable request and with appropriate justification, in accordance with institutional and ethical regulations.

## Abbreviations

CAD: Coronary artery disease
CVD: Cardiovascular disease
LDL-C: Low-density lipoprotein cholesterol
Lp(a): Lipoprotein (a)
T1D: Type 1 diabetes

## References

[1] Bhatia HS, Wandel S, Willeit P, Lesogor A, Bailey K, Ridker PM, et al. Independence of lipoprotein(a) and low-density lipoprotein cholesterol-mediated cardiovascular risk: a participant-level meta-analysis. Circulation. 2025;151(4):312–21. 10.1161/CIRCULATIONAHA.124.069556. 39492722 PMC11771346

[2] Raitakari O, Kartiosuo N, Pahkala K, Hutri-Kähönen N, Bazzano LA, Chen W, et al. Lipoprotein(a) in youth and prediction of major cardiovascular outcomes in adulthood. Circulation. 2023;147(1):23–31. 10.1161/CIRCULATIONAHA.122.060667. 36440577 PMC9797445

[3] Boffa MB, Koschinsky ML. Oxidized phospholipids as a unifying theory for lipoprotein(a) and cardiovascular disease. Nat Rev Cardiol. 2019;16(5):305–18. 10.1038/s41569-018-0153-2. 30675027

[4] Reyes-Soffer G, Ginsberg HN, Berglund L, Duell PB, Heffron SP, Kamstrup PR, et al. Lipoprotein(a): a genetically determined, causal, and prevalent risk factor for atherosclerotic cardiovascular disease: a scientific statement from the american heart association. Arterioscler Thromb Vasc Biol. 2022;42(1):e48-60. 10.1161/ATV.0000000000000147. 34647487 PMC9989949

[5] Emdin CA, Khera AV, Natarajan P, Klarin D, Won HH, Peloso GM, et al. Phenotypic characterization of genetically lowered human lipoprotein(a) levels. J Am Coll Cardiol. 2016;68(25):2761–72. 10.1016/j.jacc.2016.10.033. 28007139 PMC5328146

[6] Reyes-Soffer G, Yeang C, Michos ED, Boatwright W, Ballantyne CM. High lipoprotein(a): actionable strategies for risk assessment and mitigation. Am J Prev Cardiol. 2024;18:100651. 10.1016/j.ajpc.2024.100651. 38646021 PMC11031736

[7] Rawshani A, Sattar N, Franzén S, Rawshani A, Hattersley AT, Svensson AM, et al. Excess mortality and cardiovascular disease in young adults with type 1 diabetes in relation to age at onset: a nationwide, register-based cohort study. Lancet. 2018;392(10146):477–86. 10.1016/S0140-6736(18)31506-X. 30129464 PMC6828554

[8] Kronenberg F. Lipoprotein(a): from causality to treatment. Curr Atheroscler Rep. 2024;26(3):75–82. 10.1007/s11883-024-01187-6PubMedPMID:38252372;PubMedCentralPMCID:PMC10881767. 38252372 PMC10881767

[9] Greco A, Finocchiaro S, Spagnolo M, Faro DC, Mauro MS, Raffo C, et al. Lipoprotein(a) as a pharmacological target: premises, promises, and prospects. Circulation. 2025;151(6):400–15. 10.1161/CIRCULATIONAHA.124.069210. 39928714

[10] Harris PA, Taylor R, Thielke R, Payne J, Gonzalez N, Conde JG. Research electronic data capture (REDCap)–a metadata-driven methodology and workflow process for providing translational research informatics support. J Biomed Inform. 2009;42(2):377–81. 10.1016/j.jbi.2008.08.010. 18929686 PMC2700030

[11] Becker M, Weiskorn J, Wiegand S, Röbl M, Meissner T, Schulz E, et al. Familial hypercholesterolemia in pediatric patients with type 1 diabetes: double challenge for diagnosis and treatment. Diabetes Care. 2025;48(12):2119–26. 10.2337/dc25-1195. 41129620

[12] Li S, Hou L, Zhu S, Yi Q, Liu W, Zhao Y, et al. Lipid variability and risk of cardiovascular diseases and all-cause mortality: a systematic review and meta-analysis of cohort studies. Nutrients. 2022;14(12):2450. 10.3390/nu14122450. 35745179 PMC9231112

[13] Bangalore S, Breazna A, DeMicco DA, Wun CC, Messerli FH, TNT Steering Committee and Investigators. Visit-to-visit low-density lipoprotein cholesterol variability and risk of cardiovascular outcomes: Insights from the TNT trial. J Am Coll Cardiol. 2015;65(15):1539–48. 10.1016/j.jacc.2015.02.017. 25881936

[14] Enkhmaa B, Berglund L. Non-genetic influences on lipoprotein(a) concentrations. Atherosclerosis. 2022;349:53–62. 10.1016/j.atherosclerosis.2022.04.006. 35606076 PMC9549811

[15] Srinivasan SR, Dahlen GH, Jarpa RA, Webber LS, Berenson GS. Racial (black-white) differences in serum lipoprotein (a) distribution and its relation to parental myocardial infarction in children. Bogalusa Heart Study. Circulation. 1991;84(1):160–7. 10.1161/01.cir.84.1.160. 1829398

[16] Obisesan TO, Aliyu MH, Adediran AS, Bond V, Maxwell CJ, Rotimi CN. Correlates of serum lipoprotein (A) in children and adolescents in the United States. The third National Health Nutrition and Examination Survey (NHANES-III). Lipids Health Dis. 2004;3(1):29. 10.1186/1476-511X-3-29. 15601478 PMC544891

[17] Müller N, Schulte DM, Türk K, Freitag-Wolf S, Hampe J, Zeuner R, et al. IL-6 blockade by monoclonal antibodies inhibits apolipoprotein (a) expression and lipoprotein (a) synthesis in humans. J Lipid Res. 2015;56(5):1034–42. 10.1194/jlr.P052209. 25713100 PMC4409280

[18] Hedegaard BS, Nordestgaard BG, Kanstrup HL, Thomsen KK, Bech J, Bang LE, et al. High lipoprotein(a) may explain one-quarter of clinical familial hypercholesterolemia diagnoses in Danish lipid clinics. J Clin Endocrinol Metab. 2024;109(3):659–67. 10.1210/clinem/dgad625. 37862146

[19] Johansen AK, Bogsrud MP, Thoresen M, Christensen JJ, Narverud I, Langslet G, et al. Lipoprotein(a) in children and adolescents with genetically confirmed familial hypercholesterolemia followed up at a specialized lipid clinic. Atheroscler Plus. 2024;57:13–8. 10.1016/j.athplu.2024.06.002. 39027312 PMC11254952

[20] Nordestgaard BG, Langsted A. Lipoprotein(a) and cardiovascular disease. Lancet. 2024;404(10459):1255–64. 10.1016/S0140-6736(24)01308-4. 39278229

[21] Paré G, Çaku A, McQueen M, Anand SS, Enas E, Clarke R, et al. Lipoprotein(a) levels and the risk of myocardial infarction among 7 ethnic groups. Circulation. 2019;139(12):1472–82. 10.1161/CIRCULATIONAHA.118.034311. 30667276

