# Supplementary material for: Longitudinal variability of lipoprotein(a) in youth-onset type 1 diabetes: implications for cardiovascular risk stratification
Source: Cardiovasc Diabetol. 2026 Jun 19;25:263. doi: 10.1186/s12933-026-03252-7 (PMC13523341; doi:10.1186/s12933-026-03252-7)
Supplement: Supplementary file 1 [file 12933_2026_3252_MOESM1_ESM.docx]

**Additional file**

**Supplementary Table 1. Characteristics of study participants.**

Case subjects n=286. Results are expressed in mean ± SD and/or median and IQR (25^th^ - 75^th^), except for sex, which is expressed as n (%). Duration of diabetes was assessed at the last clinical assessment. N.M. = not measured, N.A. = not applicable.

|  | **At diabetes onset** | **At first clinical assessment** | **At last clinical assessment** | **General information** |
| --- | --- | --- | --- | --- |
| Female sex (n) | N.A. | N.A. | N.A. | 132 (46.15) |
| Annual clinical assessments (n) | N.A. | N.A. | N.A. | 4.90 ± 3.12 |
| Age at diabetes onset (years) | N.A. | N.A. | N.A. | 7.64 ± 3.82 |
|  |  |  |  | 7.57  (4.74 - 10.55) |
| Duration of diabetes (years) | N.A. | N.A. | N.A. | 6.68 ± 4.52 |
|  |  |  |  | 6.19  (2.90 - 9.58) |
| BMI (kg/m^2^) | 16.27 ± 3.53 | 18.47 ± 3.29 | 21.11 ± 3.79 | N.A. |
|  | 15.35  (14.05 - 17.33) | 17.80  (16.12 - 20.11) | 20.85  (18.26 - 23.73) |  |
| HbA1c (mmol/mol) | 101.15 ± 26.01 | 66.38 ± 15.25 | 67.45 ± 12.62 | N.A. |
|  | 100.15  (83.80-116.33) | 64.80  (57.80 -72.70) | 65.50  (59.65 - 75.40) |  |
| HbA1c (%) | 11.23 ± 2.31 | 8.20 ± 1.27 | 8.63 ± 1.57 | N.A. |
|  | 11.20  (9.80 - 12.50) | 8.00  (7.40 - 8.78) | 8.30  (7.60 - 9.30) |  |
| Lipoprotein (a) (mg/L) | N.M. | 236.93 ± 288.46 | 233.56 ± 293.68 | N.A. |
|  |  | 107.50  (41.25 - 312.75) | 108.00  (43.00 - 310.50) |  |
| Total cholesterol (mM) | N.M. | 4.35 ± 0.86 | 4.33 ± 0.90 | N.A. |
|  |  | 4.20 (3.74 - 4.90) | 4.20 (3.74 - 4.88) |  |
| LDL (mM) | N.M. | 2.40 ± 0.77 | 2.31 ± 0.73 | N.A. |
|  |  | 2.27 (1.85 - 2.82) | 2.19 (1.78 - 2.81) |  |
| HDL (mM) | N.M. | 1.68 ± 0.43 | 1.64 ± 0.38 | N.A. |
|  |  | 1.66 (1.40 - 1.91) | 1.59 (1.41 - 1.89) |  |
| Triglycerides (mM) | N.M. | 0.62 ± 0.34 | 0.80 ± 0.50 | N.A. |
|  |  | 0.54 (0.42 - 0.69) | 0.65 (0.54 - 0.90) |  |
| Insulin dose (U/kg/d) | N.M. | 0.72 ± 0.244 | 0.89 ± 0.28 | N.A. |
|  |  | 0.70 (0.57 - 0.85) | 0.86 (0.70 - 1.07) |  |

**Supplemental Figures**

**Fig. S1. Correlation between Lp(a) and HbA1c levels**

Fig.S1: Correlation between Lp(a) and HbA1c (participant median values). Each point represents one participant's median log-transformed Lp(a) and median HbA1c. Pearson and Kendall correlation coefficients are shown.

**Fig. S2: Fraction of patients doubling their Lp(a) level**

**A**

**BBBB**

Fig.S2: **A**: Intraindividual Lp(a) variability in 235 patients with ≥2 measurements. Each point represents one patient, plotted by personal minimum versus maximum observed Lp(a) value (log-scaled axes). The dashed diagonal line indicates where the minimum equals half the maximum; points below the line represent patients with a ≥2-fold intraindividual range. Overall, 75/235 patients (32%) met this criterion. Similar proportions (32–38%, accompanying table) were observed after restricting the analysis to patients with higher personal maximum Lp(a) levels (≥300, ≥500, and ≥1000 mg/L), indicating that the variability was not driven by low absolute values. Triangles: male; red symbols: female.

| **Personal maximum Lp(a) threshold (mg/L)** | **Patients above threshold (n)** | **Patients with ≥2-fold range (n)** | **Fraction** |
| --- | --- | --- | --- |
| None (full cohort) | 235 | 75 | 31.9% |
| ≥70 | 176 | 66 | 37.5% |
| ≥300 | 82 | 29 | 35.4% |
| ≥500 | 45 | 15 | 33.3% |

**B**: Each point represents one patient plotted at their personal minimum (x-axis) against their personal maximum (y-axis) Lp(a) value (both axes log-scaled). Points below the dashed line indicate a ≥ 2-fold intraindividual range. Left: patients with type 1 diabetes only (n = 214); 69/214 (32%) below the line. Right: patients with type 1 diabetes and a co-occurring autoimmune condition (Hashimoto thyroiditis or coeliac disease, n = 21); 6/21 (28%) below the line. The comparable proportions suggest that autoimmune comorbidities did not materially drive Lp(a) variability. Circles: female (red); triangles: male (teal). AID: autoimmune disease.
